# Social transition, socioeconomic status and self-rated health in China: evidence from a national cross-sectional survey (CGSS)

**DOI:** 10.3389/fpubh.2024.1359609

**Published:** 2024-06-06

**Authors:** Yi Gao, Jing Zeng, Zangyi Liao, Jing Yang

**Affiliations:** ^1^School of Public Administration, Zhongnan University of Economics and Law, Wuhan, China; ^2^School of Management, Royal Holloway, University of London, Egham, United Kingdom; ^3^School of Political Science and Public Administration, China University of Political Science and Law, Beijing, China; ^4^School of Public Administration, Hunan University, Changsha, Hunan, China

**Keywords:** China, health inequalities, social transition, self-rated health, SES, moderation mechanism

## Abstract

**Background:**

Social transition is one of the multi-level mechanisms that influence health disparities. However, it has received less attention as one of the non-traditional social determinants of health. A few studies have examined China’s social transition and its impact on health inequality in self-rated health (SRH). Therefore, this study explores the impact of China’s market-oriented reforms—social transition and socioeconomic status (SES)—on residents’ SRH.

**Methods:**

Using the cross-sectional data from the Chinese General Social Survey (CGSS) in 2017, we analyzed the effects of social transition and SES on the SRH of Chinese residents using the RIF (Recentered influence function) method. The RIF decomposition method investigated health differences among different populations and their determinants.

**Results:**

Social transition and SES have significant positive effects on the SRH of Chinese residents. The correlation between SES and the SRH of Chinese residents is moderated by social transition, implying that social transition can weaken the correlation between SES and the SRH of Chinese residents. The impacts of SES and social transition on SRH vary across populations.

**Conclusion:**

Promoting social transition and favoring disadvantaged groups with more resources are urgently needed to promote equitable health outcomes.

## 1 Introduction

Health inequalities are systematic, avoidable, and unfair differences in health outcomes observed between populations, social groups within the same population, or as a gradient across a population ranked by social position (1). Health inequalities within populations are caused by underlying structural inequalities in society. These structural inequalities produce unequal health outcomes through various socioeconomic pathways, including employment, income, housing, and educational attainment (2). Ensuring equitable access to healthcare services and support is essential for addressing health inequalities.

In the past few decades, China’s social transition process has steadily advanced, and the social economy has made considerable progress, significantly improving the living standards and health levels of Chinese residents. However, research on social transition has concentrated more on the economic sphere and its impacts on individual health (2). Most Chinese studies have considered health as a direct or indirect manifestation of class status and related resources, focusing on specific pathways and modes of action (3, 4). Few have analyzed health inequalities in China’s social transition process or the dynamic impact of SES on residents’ self-rated health (SRH) in social transition.

Social transition theory suggests that social transition brought about by market transformations includes market incentives, forces, and opportunities. The social transition will generate the appreciation of human capital and open up new channels of social mobility for Chinese residents, which will directly affect changes in individual employment and social mobility and may make individuals wait for more health resources, which affects the health and health equity of Chinese residents (5).

However, existing studies have mainly focused on the economic consequences of social transition. For example, social transition represented by market-oriented transformation has effectively contributed to the growth of regional productivity in China (6) and improved enterprise productivity and resource allocation efficiency at the micro level (7), which is conducive to the digital transformation of enterprises (8). However, the products of social transition are not exclusively economic, and existing research has paid less attention to the non-economic consequences of market transition, particularly the health of the residents. Although a few studies have examined how social transition reduces health inequalities in the non-farm labor force (9) and lowers the mental health of older adults (10), they focused on the impact of the characteristics of the market transition itself on the population’s health outcomes. The intrinsic mechanism of the relationship between SES, as the most important social determinant of health, in social transition and the changing health of the Chinese population remains unclear.

Existing literature points to a possible link between social transition, SES, and the health of the Chinese population but does not answer the key empirical question of the role of social transition in the link between SES and health. This study uses cross-sectional data from a national cross-sectional survey (CGSS) to analyze the relationship between social transition, SES, and the health of Chinese residents using interaction terms between social transition and SES. By answering the above questions, the contributions of this study are as follows: First, it analyses the relationship between market transition, SES, and Chinese residents’ SRH to better understand the role of SES in the mechanism of social transition affecting Chinese residents’ health changes. Second, it considers the heterogeneous differences in SRH among Chinese residents and fully grasps the inherent differences in the mechanism of SRH changes among Chinese residents. This is to better understand the distributional differences of social transition, SES, and SRH among Chinese residents. Third, the Oaxaca–Blinder decomposition reveals differences in SRH among different groups of Chinese residents (11–13), the source of which is social transition and SES, and suggests policy recommendations for further reducing health disparities and realizing health equity among Chinese residents.

## 2 Literature review and research hypothesis

### 2.1 Social transition and Chinese residents’ SRH

China has entered an era of rapid economic development due to social transition. The rapidly growing economy has positively impacted citizens’ health, with indicators such as life expectancy and neonatal mortality moving in a healthier direction and increased income from economic growth, leading to a more optimistic attitude toward life and positively impacting health (14, 15). According to Nee’s theory of social transition, social transition consists of market incentives, market power, and market opportunities, which, at the individual level, correspond to employment units, political capital, and social mobility (5, 16).

At the individual level, the private economy’s growth due to the social transition has narrowed the health gap between employees in the private and public sectors (9, 17). The social transition has provided more channels for upward social mobility and increased access to health resources for Chinese residents (18, 19).

### 2.2 SES and residents’ SRH

Before the 1980s, it was generally accepted in the academic community that health inequalities diminished with advances in medical technology and socioeconomic development (20, 21). However, with the publication of the Black Report, health inequalities increased (22–25). Moreover, higher social strata were advantageous for health stratification (4, 26, 27).

Most studies have used educational attainment, income level, and subjective social status (SSS) to measure SES. Many studies have explored the relationship between health and educational attainment, with the vast majority finding that educational attainment positively impacts health (28). Educational attainment reflects an individual’s ability to access resources and is considered the most important determinant of health. Income reflects a person’s spending power and ability to access healthcare resources; a large body of research supports the impact of income on health inequalities (29). Occupation reflects an individual’s social status, sense of responsibility for rights, and health risks, and there are differences in the work environment, intensity of work, and working environment among different occupational groups (30). The SSS reflects people’s social class and status and combines indicators such as income level, educational attainment, and occupational status (31). Most early studies focused on the accessibility of SSS to income and healthcare levels. Since the turn of the century, more researchers have focused on SSS’s impact on health via lifestyle and psychosocial channels (32). Compared to SES, which shows how people perceive their SES concerning others and which status group they consider themselves to belong to, SSS accurately reflects sensitive social status factors, provides scoring information compared to objective indicators, and has a larger impact on individual health (33).

### 2.3 Other factors influencing Chinese residents’ SRH

Some studies have discussed the impact of additional factors on residents’ SRH. Residents’ SRH declines with age, with higher rates of multiple morbidities in the older adult population (34). Urban–rural differences similarly impact residents’ SRH, with differences in SES resulting from urban–rural divides placing rural residents at a disadvantage in terms of SRH (35), with rural older adults having lower scores on both ADLs and IADLs (36). Marriage status is likewise an important social determinant of residents’ SRH (37), with married residents having fewer loneliness perceptions and health problems than unmarried residents in terms of health level (38). There is also a concern that residents’ SRH is influenced by religious beliefs, with participation in religious activities being a significant predictor of SRH among Christians, Muslims, and Hindus (39).

### 2.4 Conceptual framework

Social transition has contributed to China’s rapid economic growth, and rapid economic growth directly contributes to the increase in the average income level of citizens (40). Public services will tend to improve, which predicts that the health level of citizens will continue to grow with social transition. Social transition theory suggests that China’s market-oriented reforms have changed the political rights-oriented resource allocation mechanism in favor of “direct producers,” who actively participate in the market, and have weakened the privileges of “redistributors” (5). The impact of social transition on the SRH of Chinese residents is primarily manifested in three aspects: First, market transition, represented by market-oriented reforms, has provided more opportunities for higher education and continuing education, improved the self-knowledge and self-management abilities of Chinese residents, and enhanced their health literacy, which is conducive to the maintenance of a healthy state of life. Second, the favorable economic development environment created by the social transition has helped to raise the return on education and income of the Chinese residents (9, 10). This has enabled Chinese residents to have more resources to invest in their health. Third, the social transition has broadened the channels of social mobility and provided more opportunities for upward mobility, which can effectively improve the quality of life and subjective well-being of Chinese residents, leading to a more optimistic attitude toward life, which is beneficial to health (3). Therefore, this study formulates hypothesis 1.

*Hypothesis 1:* Advancement in social transition promotes the growth of SRH.

SES is one of the key determinants of health and refers to an individual’s social class or status. SES is usually measured using income, education, and occupation (41). SES is associated with health outcomes, with people of relatively low SES having a shorter life expectancy and a higher prevalence of chronic diseases than those of higher SES (42). Individuals with higher education and income are more likely to be health-advantaged (43). Unlike educational attainment and income, SSS describes how people perceive their SES about others and which status group they believe they belong to. Objective SES does not always match a person’s subjective perceived status. Low SSS is associated with a variety of physical and mental health problems, even after controlling for objective SES (33, 44). Differences in health outcomes among people of different SES can be summarized in three ways: First, there are differences in health investments among people of different SES, with those in lower SES having fewer resources to spend on staying healthy (45). Second, people with higher SES have higher health literacy and better health habits, which lead to good health status (46). Third, people of low SES are more likely to exhibit depressive tendencies than people of high SES (47). Based on this, this study formulates Hypothesis 2.

*Hypothesis 2:* SES has a significantly positive effect on SRH among Chinese residents.

The study of health disparities among Chinese residents of different SES in the context of social transition centers on how SRH disparities among these residents tend to change as the social transition progresses. Do social transition and SES affect SRH independently, or do they have a moderating effect? If social transition is not considered, the impact of SES on population health with increasing age shows a parallel effect, and when education is used as a measure of SES, some studies have found that the relationship between SES and health has not changed over time (48). The relationship between SES and Chinese residents’ SRH may change after considering social transition, an important external influence. Specifically, social transition is a process of increasing market prosperity and enriching channels of upward social mobility, and the great abundance of medical resources and explosive growth of health information brought about by social transition will affect the health outcomes of SES.

**Figure 1:**
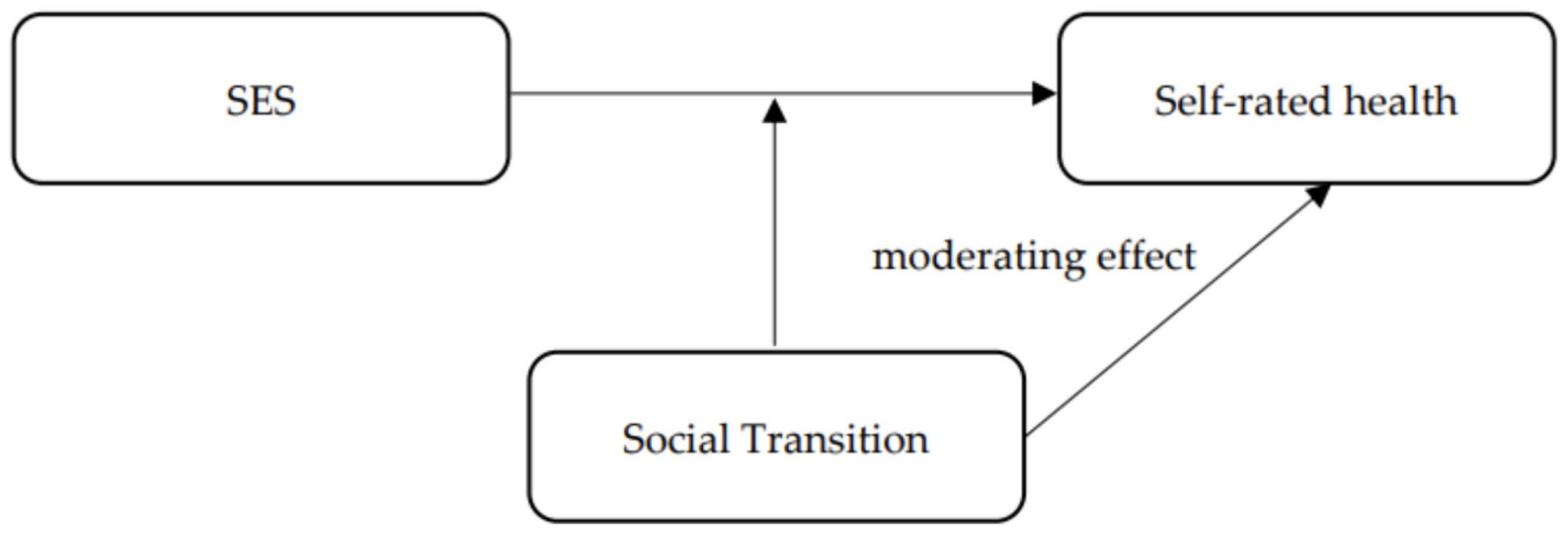
Research hypothesis.

The learning advantage of health literacy and health knowledge due to education will gradually shrink with the growth of health information brought about by the development of information technology. The Internet, as a medium for information dissemination, is characterized by fast dissemination and low cost and interacts with various health information and knowledge for users. This makes it possible to equalize information even for groups in the lower SES and facilitates access to health resources for information disadvantaged groups (49, 50). Health inequalities based on income disparities are mitigated by high rates of economic and social development brought about by social transition (51). With the balanced development of medical resources and the continuous improvement of the medical insurance system, people with lower incomes can obtain higher-quality health services. The channels for upward social mobility brought about by social transition will also make lower SES residents feel full of opportunities and maintain an optimistic mindset, thus promoting health (52). Figure 1 shows the conceptual framework of this study.

In summary, one of the distinctive features of social transition is the marked acceleration of social development and the linear growth of all types of information and resources, which can lead to greater support for people who were previously at the bottom of the social ladder. Thus, in the process of social transition, people have greater access to health-related resources and information, compensating for the health inequalities caused by SES. Accordingly, this study formulates Hypothesis 3.

*Hypothesis 3:* Social transition moderates health disparities due to SES, and the effect of SES on SRH diminishes as social transition progresses.

## 3 Methods

### 3.1 Data and statistical model

The data is taken from the 2017 Chinese General Social Survey (CGSS), the earliest national, comprehensive, and continuous academic survey project in China. The CGSS adopts a multi-stage stratified probability sampling design and conducts a continuous cross-sectional survey of more than 10,000 households in all provinces, municipalities, and autonomous regions across the country, systematically and comprehensively collecting data at the social, community, household, and individual levels of data. CGSS2017 consists of three parts: the core module, the household questionnaire module, and the social network module, which includes 783 variables. As the CGSS 2017 contains rich data on residents’ SRH-influencing factors, data from that year were selected for this study. In practice, the social transition index data were matched at the provincial level, and 11,712 samples were obtained as the final data source after eliminating invalid data for related variables. The following treatments were implemented as the study needed to explore health inequality at the overall and micro-level levels of the population.

To explore the causes and sources of health disparities among Chinese residents, this study uses a recentered influence function (RIF) regression, calculated based on the influence function (IF) and constructed by adding the original statistic to the IF. The RIF statistic has an excellent property in that its unconditional expectation is the corresponding statistic itself, laying the foundation for the RIF regression (11, 53).


$$\int RIF\left\{y_{i},v\left(F_{y}\right)\right\}=v\left(F_{y}\right)$$

An OLS regression with RIF as the explanatory variable and taking unconditional expectations on both sides of the equation gives


$$RIF\left\{y_{i},v\left(F_{y}\right)\right\}=X^{\prime }\beta +\varepsilon_{i}$$

$$v\left(F_{y}\right)=E\left[RIF\left\{y,v\left(F_{y}\right)\right\}\right]=E\left[X^{\prime }\beta \right]+E\left[\varepsilon_{i}\right]={\bar{X}}^{\prime }\beta$$

where β means that the RIF statistic for y will increase by β when the mean in the overall X increases by one unit, controlling for other things being equal. This study selected the mean, variance, and quantile distance as the v -statistic.

The RIF regression model is implemented in two steps. Step 1: Calculate the RIF value of Chinese residents’ SRH, denoted RIF_SRH, which measures health disparities. Second, the RIF estimate of SRH is the explanatory variable in the OLS regression to obtain the RIF_OLS regression model. The simplified form is as follows:


$$RIF:\mathrm{self}:\mathrm{ratedhealth}=a_{0}+\sum \beta_{i}X_{i}+\varepsilon_{i}$$

RIF:self:ratedhealth denotes the RIF statistics of individual SRH status, $a_{0}$ is the intercept term, $X_{i}$ is the social transition, SES, and control variables, the corresponding coefficients to be estimated are β , and $\varepsilon_{i}$ is the random disturbance term. Compared to the OLS model, the RIF_OLS model more intuitively reflects health disparities among Chinese residents.

To explore the moderating effect of social transition on the relationship between SES and SRH among Chinese residents, this study designed the following model:


$$\begin{array}{c} \mathrm{Self}\_\mathrm{ratedhealt}h_{i}=\alpha_{0}+\alpha_{1}\mathrm{social}\;\mathrm{transitio}n_{i}\\ +\;\alpha_{2}SES_{i}+\alpha_{3}Z_{i}\ldots +\alpha_{k}X_{i}+\varepsilon_{i} \end{array}$$

Subsequently, the model above was designed. The variable $\mathrm{social}\;\mathrm{transitio}n_{i}$ indicates the provincal degree of social transition. $Z_{i}$ indicates the interaction term between SES and social transition. $X_{i}$ indicates the control variable.

This study also used RIF and Oaxaca–Blinder decompositions to analyze the reasons for the emergence of health stratification.


$$\begin{array}{l} v_{i}=E\left[RIF\left(y,v\left(F_{Y|T=1}\right)\right)\right]={\bar{X}}^{1\prime }{\hat{\beta }}^{1}v_{0}=E\left[RIF\left(y,v\left(F_{Y|T=0}\right)\right)\right]\\ \;={\bar{X}}^{0\prime }{\hat{\beta }}^{0}v_{C}={\bar{X}}^{1\prime }{\hat{\beta }}^{0} \end{array}$$

$v_{C}$ refers to the counterfactual group statistics. To ensure the accuracy of the construction, the distribution fit was constructed using a reweighting adjustment that can be constructed from the logit model, after which an RIF regression was performed to obtain the coefficient estimates:


$$F_{Y}^{C}=\int F_{Y|X,T=0}dF_{Y|X,T=1}≅\int F_{Y|X,T=0}dF_{X|T=0}\omega \left(X\right)$$

$$v_{c}=E[\left[RIF\left(y,vF_{Y}^{C}\right))\right]={\bar{X}}^{C\prime }{\hat{\beta }}^{C}$$

Immediately following the RIF decomposition, the decomposition process is as follows: the first two terms are coefficient effects, the last two are characteristic effects, and the third is a pure characteristic effect. Using RIF decomposition, this study decomposes the RIF statistics using the SRH composition of Chinese residents into a component that can be explained by social transition and SES and a component that cannot.


$$\Delta v={\bar{X}}^{1}\left({\hat{\beta }}_{1}-{\hat{\beta }}_{C}\right)+\left({\bar{X}}^{1}-{\bar{X}}^{C}\right){\hat{\beta }}_{C}+\left({\bar{X}}^{C}-{\bar{X}}^{0}\right){\hat{\beta }}_{0}+{\bar{X}}^{C}\left({\hat{\beta }}_{C}-{\hat{\beta }}_{0}\right)$$

### 3.2 Variables description

#### 3.2.1 SRH status

To examine individual health status and its influencing factors, this study used the CGSS questions on SRH status to measure health. SRH status is an important health assessment tool and a subjective indicator of SRH status. It is an important predictor of morbidity and mortality, even when demographic and chronic disease characteristics are controlled, and is valid across other studies (54).

#### 3.2.2 Social transition

To measure the degree of social transition, we mainly relied on the marketization index of Chinese provinces. Some scholars used the marketization index data from reports to study issues related to China’s social transition (55–57), demonstrating the indicator’s reliability and validity in studying China’s social transition. In this study, the marketization indices of different provinces were matched to the corresponding samples. The process of social transition is a social reform process that involves all aspects of society. Fan’s use of the five-factor analysis system of measurement is more relevant to the content of this study, which comprehensively measures the relative situation of the marketization process of each province in terms of the relationship between the government and the market, the product market, the factor market, the market intermediary, the legal and institutional environment, and the development of the non-state economy, among others, and then derives the marketization index (55). This study chose the perspective of marketization to discuss the relationship between SES and SRH of Chinese residents in the process of social transition, which is also based on the following two considerations: First, after the reform and opening up, the market economy has had an increasingly prominent impact, bringing more employment opportunities and opening up social mobility channels in China, and the SES and economic status of Chinese residents have been significantly changed. This makes marketization a crucial perspective for analyzing health stratification; second, marketization makes an essential driving force for social transition, and changes in residents’ SRH are a product of social transition; thus, exploring the impact of marketization on China’s residents’ SRH can help to reflect from the side how social transition works on residents’ SRH.

#### 3.2.3 SES

SES was measured using years of education, income, and subjective social status (SSS). Years of education were measured according to the highest educational attainment of the respondents (No schooling = 1; Elementary school = 6; Junior high school = 9; High school = 12; Junior college = 15; Bachelor’s degree and above = 16). Income was measured according to the respondent’s total annual income and taken as the natural logarithm. SSS was measured according to the social class in which the respondents perceived it (Low = 1; Middle = 2; High = 1).

#### 3.2.4 Control variables

In this study, the control variables were categorized into three groups based on reference to existing studies. First, we controlled for variables that could lead to bias in social transition during data analysis, including respondents’ gender (male = 1), age and the square term of age, the residential areas (rural = 1; urban = 0), marriage status (1 = married; unmarried and other = 0), and frequency of exercise over 30 min per week (58–61). In the context of the era of information technology, and considering that the use of information technology may have a certain impact on the use of medical resources and the search for health information by Chinese residents (62), Internet use by respondents was controlled. Table 1 presents the variable assignment table and descriptive statistics.

**Table 1:** Descriptive statistics of main variables (*N* = 11,712).

Variable type	Variable name		Variable interpretation	Mean	SD
Dependent variable	Health status	Self-rated health	Very unhealthy = 1; Rather unhealthy = 2; Fair = 3; Healthy = 4; Very healthy = 5	3.457	1.100
Independent Variables	SES	Educational attainment	No schooling = 0; Elementary school = 6; Junior high school = 9; High school = 12; Junior college = 15; Bachelor’s degree and above = 16	8.998	4.723
		Income	Income is taken as the logarithm	8.350	3.833
		SSS	Low = 1; Middle = 2; High = 3	1.457	0.595
	Social transition	Market transition index	China’s Provincial Marketisation Index	8.847	1.342
Control variables	Individual features	Sex	Female = 1; Male = 0	0.475	0.499
		Age	Actual age (years)	51.228	16.717
		Age^2	Age squared	2903.745	1734.541
		Residential areas	Rural = 1; urban = 0	0.540	0.498
		Marriage status	Married = 1; Unmarried and Other = 0	0.773	0.419
		Exercise	Values range from 1 to 5, with higher values indicating higher exercise frequency	2.150	1.556
		Internet	Values range from 1 to 5, with higher values indicating higher frequency of using the internet	2.800	1.720

## 4 Results

### 4.1 Impact of social transition on the SRH of the Chinese residents

The first three columns of Table 2 show the effect of social transition on the mean SRH of the Chinese residents. Model 1 contains only social transition and control variables, and the estimation results show that social transition is significantly and positively associated with Chinese residents’ SRH. Model 2 contains only SES variables and control variables, and the results show that educational attainment, income, and SSS are also significantly and positively associated with the SRH of Chinese residents. Model 3 put in social transition, SES, and control variables simultaneously, and the analysis results also show that social transition, SES, and Chinese residents’ SRH present a significant positive relationship. Therefore, Hypotheses 1 and 2 were verified.

**Table 2:** Impact of social transition on the SRH among Chinese residents (*N* = 11,712).

Variables	Model 1	Model 2	Model 3
	SRH	SRH	SRH
Social transition	0.062***		0.047***
	(0.007)		(0.007)
Educational attainment		0.016***	0.015***
		(0.003)	(0.003)
Income		0.023***	0.022***
		(0.003)	(0.003)
SSS Compared to low SSS (low = 1)			
Middle SSS		0.290***	0.286***
		(0.019)	(0.019)
High SSS		0.395***	0.385***
		(0.038)	(0.038)
Sex	0.121***	0.073***	0.080***
	(0.018)	(0.018)	(0.018)
Age	−0.046***	−0.046***	−0.046***
	(0.003)	(0.003)	(0.003)
Age^2	0.000***	0.000***	0.000***
	(0.000)	(0.000)	(0.000)
Residential areas	−0.094***	−0.000	0.024
	(0.020)	(0.022)	(0.022)
Marriage status	0.144***	0.101***	0.105***
	(0.024)	(0.024)	(0.024)
Exercise	0.071***	0.062***	0.061***
	(0.006)	(0.006)	(0.006)
Internet	0.087***	0.066***	0.059***
	0.121***	0.073***	0.080***
Cons	4.043***	4.164***	3.802***
	(0.106)	(0.095)	(0.110)
Avg. RIF	3.457	3.457	3.457
N	11,712	11,712	11,712
r2	0.213	0.238	0.241

The parentheses are standard errors; ^∗∗∗^, ^∗∗^, and ^∗^ indicate significance at the 1, 5, and 10% levels, respectively.

Additionally, the results of the analysis of control variables illustrate. Compared with women, men’s SRH was significantly higher, there was an inverted U-shaped relationship between age and Chinese residents’ SRH, and married residents’ SRH was significantly higher than that of unmarried residents. Exercise frequency significantly and positively affects SRH in Chinese residents. Internet use is also significantly positively associated with SRH, suggesting the existence of a digital health divide.

### 4.2 Moderating effect of social transition on SES and SRH of Chinese residents

**Figure 2:**
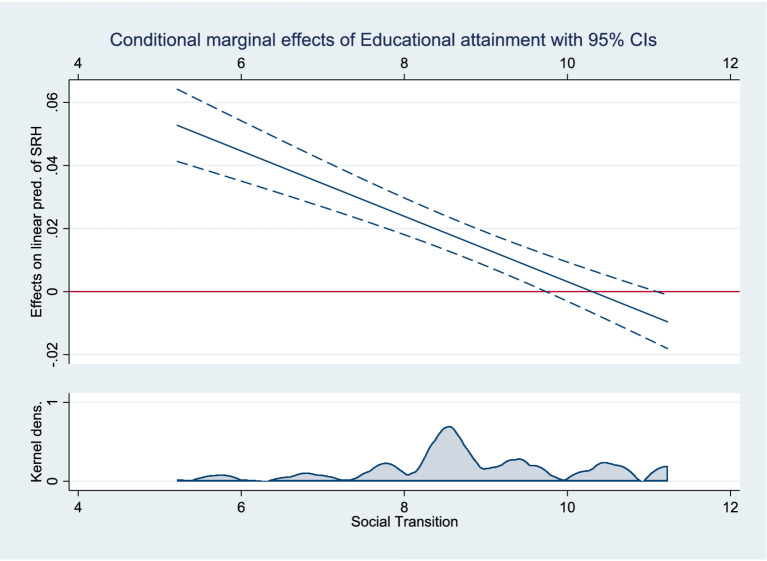
Moderating effects of social transition on educational attainment and SRH.

**Figure 3:**
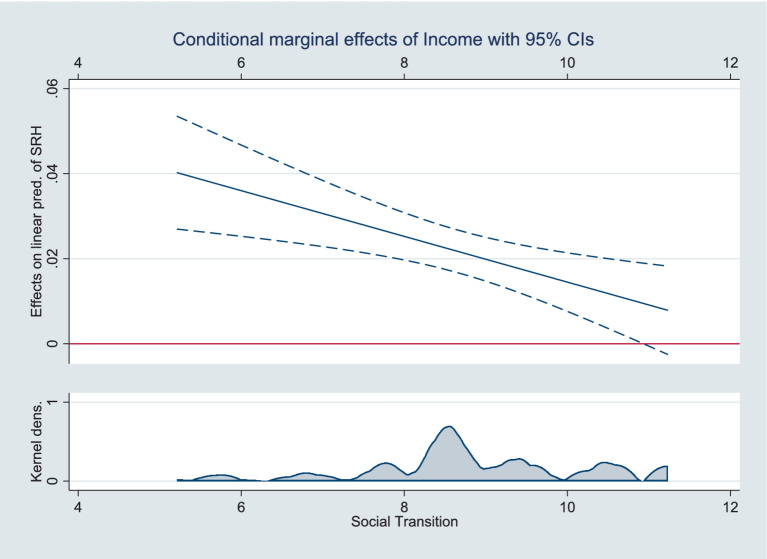
Moderating effects of social transition on income and SRH.

**Figure 4:**
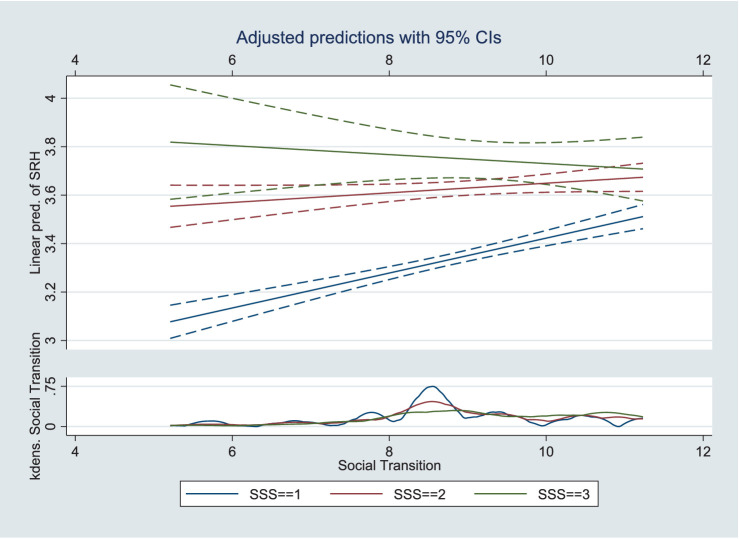
Moderating effects of social transition on SSS and SRH.

Three models were designed to test Hypothesis 3: Table 3 shows the moderating effect results. Models 1, 2, and 3 include interaction terms for social transition and educational attainment, income, and SSS, respectively. The analysis showed that the cross-multiplication terms were significant and all were opposite the sign of the main effect, indicating that the moderating effect held. Figures 2–4 present the results of the analysis of the moderating effect more intuitively. As shown in Figure 2, the effect of educational attainment on Chinese residents’ SRH diminished as the degree of social transition continued to increase, and the effect is progressively insignificant, indicating that the correlation between educational attainment and SRH diminished as social transition advanced. Figure 3 shows the results of the same analyses, where the correlation between the logarithm of income and the SRH of Chinese residents diminished with the advancement of social transition. Figure 4 shows how the three different SSS change with social transition. The analyses show that the correlation between middle and low SSS residents and SRH strengthens with the advancement of social transition; the difference in SRH between Chinese residents with different SSS will gradually decrease. Therefore, Hypothesis 3 was supported.

**Table 3:** Influence and moderating effect of SES on SRH of Chinese residents (*N* = 11,712).

Variables	Model1	Model 2	Model 3
	SRH	SRH	SRH
Social transition	0.145***	0.094***	0.120***
	(0.015)	(0.017)	(0.018)
Educational attainment	0.107***	0.015***	0.015***
	(0.013)	(0.003)	(0.003)
Income	0.021***	0.068***	0.021***
	(0.003)	(0.016)	(0.003)
SSS Compared to low SSS (low = 1)			
Middle SSS	0.281***	0.284***	0.719***
	(0.019)	(0.019)	(0.100)
High SSS	0.395***	0.386***	1.281***
	(0.041)	(0.041)	(0.206)
Educational attainment* social transition	−0.010***		
	(0.001)		
Income* social transition		−0.005***	
		(0.002)	
SSS* social transition			−0.049***
			(0.011)
Cons	2.907***	3.401***	3.601***
	(0.167)	(0.177)	(0.122)
Control	YES	YES	YES
*N*	11,712	11,712	11,712
r2	0.244	0.241	0.242

The parentheses are standard errors; ^∗∗∗^, ^∗∗^, and ^∗^ indicate significance at the 1, 5, and 10% levels, respectively. Control means the control variables.

### 4.3 Heterogeneity analysis among different groups

To validate the robustness of the model results, we constructed and re-estimated the model using subsamples of data from rural, urban, older adults, non-older adults, urban older adults, rural older adults, male older adults, and female older adults. Table 4 shows the results. The effects of social transition and the three variables measuring SES on SRH remained significant in most subsamples.

**Table 4:** Robustness test results (*N* = 11,712).

Variables	Model 1	Model 2	Model 3	Model 4	Model 5	Model 6	Model 7	Model 8
	Rural	Urban	Older adult	Non-older adult	Rural older adult	Urban older adult	Male older adult	Female older adult
Social transition	0.083***	0.015	0.049***	0.047***	0.094***	0.025	0.081***	0.018
	(0.011)	(0.009)	(0.013)	(0.008)	(0.023)	(0.016)	(0.019)	(0.019)
Educational attainment	0.024***	0.007*	0.013***	0.017***	0.022***	0.007	0.012*	0.015**
	(0.004)	(0.004)	(0.004)	(0.004)	(0.007)	(0.006)	(0.007)	(0.006)
Income	0.024***	0.017***	0.027***	0.018***	0.027***	0.027***	0.037***	0.022***
	(0.003)	(0.004)	(0.005)	(0.003)	(0.007)	(0.009)	(0.008)	(0.007)
SSS Compared to low SSS (low = 1)								
Middle SSS	0.314***	0.254***	0.294***	0.275***	0.372***	0.223***	0.277***	0.309***
	(0.028)	(0.026)	(0.035)	(0.023)	(0.055)	(0.045)	(0.051)	(0.049)
High SSS	0.445***	0.360***	0.329***	0.413***	0.387***	0.283***	0.196**	0.467***
	(0.073)	(0.048)	(0.071)	(0.050)	(0.137)	(0.081)	(0.096)	(0.105)
Empirical P-values	−0.068***		−0.002		−0.069***		−0.063**	
Control	YES	YES	YES	YES	YES	YES	YES	YES
Cons	3.493***	4.071***	2.657**	3.762***	2.205	3.361**	1.233	4.388**
	(0.169)	(0.142)	(1.297)	(0.179)	(2.099)	(1.635)	(1.882)	(1.811)
N	6,322	5,390	4,112	7,600	2098	2014	1997	2,115
r2	0.251	0.208	0.113	0.186	0.085	0.094	0.120	0.097

The parentheses are standard errors; ^∗∗∗^, ^∗∗^, and ^∗^ indicate significance at the 1, 5, and 10% levels, respectively. Control means the control variables. Empirical *p*-values are the results of the Fischer test.

The results of the analysis of Models 1 and 2 show that when all other conditions have been controlled, social transition only significantly and positively affected rural residents’ SRH. This indicates that there are still differences in the process of social transition in rural areas and that rural areas with a high degree of social transition have a more rapid development of the level of medical technology and a greater rationing of health resources. Consequently, the health of the population in rural areas, where the process of social transition is more rapid, is better.

The analysis results of Model 3 and Model 4 show that social transition is significantly related to the SRH of older adults, and the results of the Fisher’s Permutation test showed that social transition contributed to SRH in older adults no differently than in non-older adults. It indicates that older adults, as a vulnerable group, have received sufficient attention in the process of social transition, and the development of smart medical care has created conditions for older adults to enjoy convenient medical resources and improve their health capital.

As a health-vulnerable group, the physical functioning of the older adult group declines gradually with age. To further analyze the heterogeneous differences within the older adults, this study continued to differentiate the older adults into rural older adults, urban older adults, male older adults, and female older adults. The results of Model 5 and Model 6 show that social transition has a significant positive effect on the SRH of rural older adults, while it has no significant effect on the SRH of urban older adults. This indicates that the equalization of basic public health services brought about by social transition has enabled rural older adults to enjoy high-quality healthcare resources and gradually narrowed the health gap with urban older adults. Simultaneously, it also shows that the problem of uneven development still exists in rural areas, and access to medical resources in rural areas with a higher degree of transition is more convenient than that in rural areas with a lower degree of transition, which in turn creates health differences among the older adults in rural areas. The coefficient of influence of social transition on the SRH of male older adults is greater than that of female older adults. This shows that there are still sex differences in the health dividends brought about by social transition, and female older adults, as a vulnerable group, need more healthcare and resources.

To more clearly analyze the health differences among different groups of Chinese residents and test the robustness of the heterogeneity analysis, this study continued with Fisher’s permutation test. The significance of differences in social transition coefficients between groups was tested using an empirical *p*-value obtained from a 1,000-bootstrap sampling. The empirical *p*-values obtained using the bootstrap method further validated the statistical significance of these differences. Significant differences in the coefficients of social transition exist between rural and urban, rural and non-rural older adults, and male and female older adults. This finding suggests that the effect of social transition on the SRH of the Chinese population varies among different groups.

To further investigate the sources of health differences among different groups of Chinese residents, this study used the Oaxaca–Blinder decomposition and obtained the characteristic effects (explainable part) and coefficient effects (unexplainable part) of the mean SRH of Chinese residents in different groups through RIF regression.

The results of this analysis are presented in Table 5. Group 1 represents rural, non-older adults, urban older adults, and older adult women. The differences among the different groups of Chinese residents’ health were significant. By disentangling the characteristic and coefficient effects, the degree of contribution of different explanatory variables to health differences among different groups of Chinese residents can also be obtained. The contribution of the characteristic effects of the social transition to the health differences of different groups of Chinese residents are 26.15, 1.09, 13.95, and 3.33%, respectively. Specifically, the high percentage of health disparities between urban and rural residents and between urban older adults and rural older adults is explained by social transition, indicating that attention should be paid to the development imbalance among regions in the subsequent development process. The characteristic effects of educational attainment, income, and SSS also contributed significantly to health differences among the different groups of Chinese residents. Therefore, although social transition has a positive net effect on the health of Chinese residents, there is still a need to focus on balancing regional development and achieving health equity. In addition, through the Oaxaca–Blinder decomposition, SES remains an important social determinant of health stratification among this population.

**Table 5:** Results of the Oaxaca–Blinder decomposition through RIF regression (*N* = 11,712).

Variables	Model 1	Model 2	Model 3	Model 4
	Rural	Older adult	Rural_older adult	Female_older adult
Group_1	3.597***	3.715***	3.175***	2.879***
	(0.014)	(0.012)	(0.022)	(0.023)
Group_c	3.831***	3.111***	3.175***	3.018***
	(0.021)	(0.028)	(0.046)	(0.028)
Group_2	3.338***	2.981***	2.795***	3.089***
	(0.015)	(0.017)	(0.024)	(0.024)
Difference	0.260***	0.734***	0.380***	−0.210***
	(0.020)	(0.020)	(0.033)	(0.033)
ToT_Explained	0.493***	0.131***	0.380***	−0.070***
	(0.019)	(0.021)	(0.042)	(0.014)
ToT_Unexplained	−0.234***	0.604***	0.000	−0.139***
	(0.026)	(0.032)	(0.053)	(0.036)
**Explained**				
Total	0.493***	0.131***	0.380***	−0.070***
	(0.019)	(0.021)	(0.042)	(0.014)
Pure_explained	0.559***	0.119***	0.355***	−0.075***
	(0.021)	(0.010)	(0.046)	(0.013)
Specif_err	−0.066***	0.011	0.025	0.005
	(0.013)	(0.015)	(0.028)	(0.004)
**Pure_explained**				
Social transition	0.068***	−0.008***	0.053***	0.007***
	(0.010)	(0.002)	(0.017)	(0.002)
Educational attainment	0.093***	0.044***	0.072***	−0.022*
	(0.017)	(0.011)	(0.027)	(0.012)
Income	0.051***	0.018***	0.069***	−0.034***
	(0.008)	(0.003)	(0.020)	(0.008)
SSS	0.054***	0.009***	0.089***	−0.003
	(0.006)	(0.002)	(0.021)	(0.002)

The parentheses are standard errors; ^∗∗∗^, ^∗∗^, and ^∗^ indicate significance at the 1, 5, and 10% levels, respectively.

## 5 Discussion

Overall, social transition had a significant positive impact on the SRH of Chinese residents, consistent with the results of previous studies (9). This study uses the marketization index to measure social transition in China. The Marketization Index is dynamic, and using this indicator to measure social transition in China confirms the stability of the impact of social transition on individual health. In other studies, scholars have used age-period cohorts to measure social transition and concluded that a good social environment is associated with good health (63).

This study also found that SES is an important social determinant of Chinese residents’ SRH. Objective SES, represented by education and income, has a significant positive effect on Chinese residents’ SRH (64–66), SSS also has a significant positive effect on Chinese residents’ SRH (67) and expectations of a better future life as a result of social transition and improved attitudes toward social change positively affect Chinese residents’ SRH (56). These findings reaffirmed the strong positive relationship between SES and SRH. Simultaneously, this study also found that differences in SES are determinants of health inequality among Chinese residents. The cumulative effect of SES advantages/disadvantages affects Chinese residents’ SRH stratification.

This study found social transition can narrow SRH disparities due to SES. SES is considered a crucial mediating factor that affects health. For example, in research on the impact of children’s SES on health and healthy behaviors in adulthood, adult SES was considered a mediating factor (68). However, in this study, the social transition was an external environmental factor and a macro change in Chinese residents’ social and living environments. The growth of health information brought about by social transition may increase the methods and efficiency of Chinese residents in receiving health information, thereby improving health literacy. Health literacy improvement is an important moderating factor for SES and health outcomes (69). Therefore, changes in the living environments of Chinese residents brought about by social transition have an impact at the individual level, leading to moderating effects.

Regarding other influences on SRH, consistent with previous studies, sex differences in SRH exist, and women are more likely than men to perceive their SRH as worse (70, 71). This study also found that the SRH of Chinese residents deteriorates with age, confirming the existence of an age-period cohort effect on health. This study also found that Chinese residents’ SRH deteriorated with age, confirming the existence of an age–cohort effect on health (63).

This study found differences in the health-promoting effects of social transition on different groups of Chinese residents through Oaxaca–Blinder decomposition. The health differences between rural and urban, older adults and older adults, rural older adults and urban older adults, male older adults, and female older adults stem in part from social transition. Therefore, in the process of social transition, attention should be paid to protecting the health of vulnerable groups and providing them with health assistance. Family doctors and community doctors are encouraged to use information technology to provide health services to vulnerable groups, guide them to improve their health literacy, and increase the utilization rate of health services.

Although this study reached the above conclusions, it also has the following shortcomings: First, this study measures residents’ health mainly using SRH indicators, and although SRH indicators have good reliability and validity (54), there has been no discussion on objective health indicators or mental health. The advancement of social transition leads to changes in residents’ social mentalities. In urban areas with a high degree of social transition, rapid changes in the social environment may lead to anxiety and other emotions. Therefore, the impact of marketization on residents’ mental health should be explored in future research. Second, in the rapidly developing marketization process, differences in the ability to use information technology have become a social determinant; the higher the degree of marketization development, the higher the degree of penetration of smart healthcare, and whether differences based on SES will affect residents’ ability to use information technology, thus making the socioeconomically disadvantaged groups unable to enjoy smart healthcare services in a more disadvantageous position (72), is worthy of further exploration.

Therefore, during subsequent research, the measurement system for residents’ health should be further enriched, beginning with more multidimensional indicators to measure the level of residents’ health and its differences accurately. Second, in the social context of aging and intertwined information technology, subsequent studies should further focus on the health differences brought about by the uneven development of information technology, digitalization, and the digital economy and whether the SES differences will be extended to the differences in the ability to use information technology and produce uneven access to healthcare and health resources, which will in turn result in health stratification based on differences in the ability to use information technology. Third, this study used cross-sectional data for analysis, which makes it difficult to observe the long-term effects of market transformation and SES on health over the life course of Chinese residents. It should be analyzed using time-series data in future studies.

## 6 Conclusion

Using data from the China General Social Survey in 2017 (CGSS2017), this study empirically analyzed the effects of social transition, and SES on Chinese residents’ SRH and differences in heterogeneity using RIF regression. It also explored the moderating role of social transition in the impact of SES on Chinese residents’ SRH and drew the following conclusions:

First, social transition had a significant positive effect on Chinese residents’ SRH. China’s economic acceleration induced by market-oriented reforms has greatly enriched the development of the health industry, with significantly higher mean scores for residents’ SRH in regions with higher degrees of social transition. Second, SES had a significant positive effect on the SRH of Chinese residents. Higher educational attainment, income, and SSS led to a significant increase in residents’ SRH. Third, health stratification caused by SES gradually narrowed with the advancement of social transition. Social transition can enrich the supply channels of health resources, reducing health inequities and thus weakening social stratification due to SES. The results of the Oaxaca–Blinder decomposition suggest that social transition and SES differences are still sources of SRH disparities among Chinese residents, and more attention should be paid to balanced development and the health needs of disadvantaged groups in the subsequent development process.

This study found that social transition, as a non-traditional social determinant, can have a differential impact on the health of the Chinese population. The influence of social transition on health disparities among Chinese residents has multi-level mechanisms. Therefore, to reduce health inequalities among Chinese residents, public health policies should be optimized in terms of social transition and SES, with the following specific recommendations:

First, the social transition should be continuously promoted to enhance the overall level of Chinese residents’ SRH. The emphasis should be on promoting the development of the medical resources market, diverting the medical and healthcare consumption of Chinese residents through the dual regulation of administrative and market means, stimulating the health demand of Chinese residents, and improving the level of health at multiple levels. Promote medical science and technology innovation to provide strong support for improving the health of Chinese residents. Ensure that the rapid economic growth brought about by the continued advancement of social transition will lay a solid foundation for maintaining people’s health, and upgrading the consumption structure will create a broad space for developing health services.

Second, the government should strengthen policy formulation and implementation. It should establish a system of regulations to ensure that vulnerable groups have equal access to healthcare resources and increase efforts to formulate and implement health policies. Simultaneously, it should strengthen the regulation of medical services to ensure reasonable distribution.

Finally, more health resources should be provided to vulnerable groups. A mechanism should be established for the balanced regional development of health services, guiding the rational flow of medical resources, and reducing the imbalance of development between urban and rural. The government should strengthen the training and equipping of medical professionals in underdeveloped regions. Health services for vulnerable groups should be further improved. Family and community doctors should be trained to meet the health needs of key populations, particularly rural older adults and female older adults. Increase the frequency of health knowledge campaigns to help vulnerable groups improve their health literacy, and conduct regular health education at the community level to help them develop healthy habits and promote health.

## Data availability statement

The raw data supporting the conclusions of this article will be made available by the authors, without undue reservation.

## Ethics statement

The study was conducted in accordance with the Declaration of Helsinki.

## Author contributions

YG: Writing – original draft, Writing – review & editing. JZ: Conceptualization, Investigation, Project administration, Supervision, Writing – review & editing. ZL: Formal analysis, Funding acquisition, Project administration, Resources, Validation, Writing – review & editing. JY: Conceptualization, Data curation, Formal analysis, Funding acquisition, Investigation, Methodology, Project administration, Resources, Software, Supervision, Validation, Visualization, Writing – original draft.
